# Niemann-Pick C2 Proteins: A New Function for an Old Family

**DOI:** 10.3389/fphys.2018.00052

**Published:** 2018-01-31

**Authors:** Jiao Zhu, Mengbuo Guo, Liping Ban, Li-Mei Song, Yang Liu, Paolo Pelosi, Guirong Wang

**Affiliations:** ^1^State Key Laboratory for Biology of Plant Diseases and Insect Pests, Institute of Plant Protection, Chinese Academy of Agricultural Sciences, Beijing, China; ^2^Department of Grassland Science, College of Animal Science and Technology, China Agricultural University, Beijing, China

**Keywords:** Niemann-Pick protein C2, Lepidoptera, *Helicoverpa armigera*, *in situ* hybridization, immunocytochemistry, ligand-binding, gossypol

## Abstract

Niemann-Pick proteins type C2 (NPC2) are carriers of cholesterol in vertebrates, with a single member in each species. The high sequence conservation between mammals and across vertebrates is related to their common function. In contrast, NPC2 proteins in arthropods have undergone extensive duplication and differentiation, probably under environmental pressure, and are likely to have different functions. Recent studies have suggested that in arthropods these proteins might act as carriers for semiochemicals and other hydrophobic compounds. In this study we focused on the function of a specific NPC2 gene in the moth *Helicoverpa armigera* (HarmNPC2-1). This protein binds several flavonoids with micromolar dissociation constants. The best ligand was gossypol, present in cotton, one of the main host plants for *H. armigera*. Western blot revealed the presence of HarmNPC2-1 in different parts of the body, including the antennae, proboscis, and abdomen. In the antennae, *in situ* hybridization experiments produced strong staining in auxiliary cells at the base of sensilla trichodea, basiconica, coeloconica, and chaetica. Immunocytochemistry confirmed the expression of the protein in sensilla chaetica. Our results support a role of semiochemical carriers for NPC2 proteins in insects and indicate such proteins as new targets for insecticide-free pest population control.

## Introduction

Niemann-Pick proteins of class C2 (NPC2) are present in all vertebrates, where they act as carriers for cholesterol and lipids. Serious diseases are related to their absence or malfunctioning (Bjurulf et al., 2008; Griese et al., 2010; Balboa et al., 2012; Frolov et al., 2013). A single gene is generally expressed in each vertebrate species (Storch and Xu, 2009) with identical amino acids of about 75% among mammals and of 55–70% across vertebrate classes. Such high conservation may be related to their affinity for the same ligands (Pelosi et al., 2014).

In arthropods, from Chelicerata and Crustacea to Insecta, we find several genes encoding NPC2 proteins in each species. These genes, unlike those of vertebrates, are highly divergent both within and between species (Pelosi et al., 2014, 2017; Vizueta et al., 2016; Zhu et al., 2016), suggesting that such extensive duplication and differentiation may have probably occurred in arthropods under environmental pressure (Pelosi et al., 2014). These facts, together with their binding affinity for small hydrophobic compounds led to the hypothesis that NPC2 proteins could represent a third class of binding proteins for semiochemicals in arthropods (Pelosi et al., 2014). Such idea was further supported by the identification of several NPC2 genes in chemosensory organs of the spider *Dysdera silvatica* and other crustacea and chelicerata (Vizueta et al., 2016), as well as by the detection of NPC2 proteins in chemosensory structures of the ant *Camponotus japonicus* (Ishida et al., 2014) and the ticks *Ixodes ricinus* (Iovinella et al., 2016) and *Amblyomma americanum* (Renthal et al., 2016).

Interestingly, NPC2 proteins share some structural and functional characteristics with odorant-binding proteins (OBPs) and chemosensory proteins (CSPs), soluble polypeptides involved in detection and delivery of semiochemicals in vertebrates and in insects (Vogt and Riddiford, 1981; Pelosi et al., 1982, 2017; Angeli et al., 1999). As OBPs and CSPs, also NPC2 proteins present a signal peptide revealing their secretory nature. Concerning OBPs, those of vertebrates are markedly different from those of insects in terms of amino acid sequence and three-dimensional folding (Pelosi, 1994; Tegoni et al., 2000), despite their common name. The first belong to the family of lipocalins and have the typical β-barrel structure, made of eight β-sheets and a short α-helical segment (Bianchet et al., 1996; Tegoni et al., 1996). The OBPs of insects, in contrast, are made of α-helical domains connected by short unstructured loops and held together in a compact structure by three conserved disulphide bridges (Sandler et al., 2000). Proteins of both classes are extremely compact and stable, and this characteristic is also shared with NPC2 proteins. Moreover, the three-dimensional folding of NPC2 (Friedland et al., 2003; Ishida et al., 2014) proteins resembles the β-barrel of lipocalins, while the presence of six conserved cysteines paired in three disulphide bridges reminds of insect OBPs.

Both vertebrate and insect OBPs are also widely and abundantly found in body fluids, where they are bound to specific pheromones and likely control their delivery in the external environment. Saliva, urine, pheromone glands, seminal fluid, and other biological secretions have been reported to contain OBPs in high concentrations (Marchese et al., 1998; Cavaggioni and Mucignat-Caretta, 2000; Iovinella et al., 2011; Sun et al., 2012; Beynon et al., 2014; Mastrogiacomo et al., 2014; Pelosi et al., 2017).

In this study, we have expressed a member of *Helicoverpa armigera* NPC2 in a bacterial system, measured its ligand-binding properties and monitored its expression in different parts of the body, using *in situ* hybridization and immunocytochemistry, in order to explore a putative role of semiochemical carrier for these proteins in insects.

## Materials and methods

### Insects

Cotton bollworm *H. armigera*, were reared at the Institute of Plant Protection, Chinese Academy of Agricultural Sciences, Beijing, China. Antennae and other parts of the body were collected from 3-day-old moths and immediately used or kept at −70°C.

### Reagents

All enzymes, unless otherwise stated, were from Thermo Scientific. Oligonucleotides were custom synthesized and plasmids were sequenced at Sheng Gong, Beijing, China. Urea, Tris, glycine, and other common reagents were from Amresco, Solon, USA. Flavonoids were purchased from J&K Chemical ltd, Shanghai, China. All other ligands were from Sigma-Aldrich and of reagent grade.

### RNA extraction and cDNA synthesis

Total RNA was extracted from antennae using Trizol Reagent (Invitrogen, Carlsbad, CA) along with following the manufacturer's instructions. The first-strand cDNA was synthesized from 2 μg of total RNA using an oligo-dT primer and the Revert Aid First Strand cDNA Synthesis Kit (Fermentas, Glen Burnie, MD) and following the manufacturer's protocol. The product was either used directly for PCR amplification or stored at −70°C.

### Bacterial expression and purification of HarmNPC2-1

For the expression of HarmNPC2-1 (acc no. XP_021184351), the coding region of the mature protein sequence was amplified by PCR using specific primers encoding the first and the last six amino acids: HarmNPC2-1-Nde AACATATGAAATACTACACGGATTG; HarmNPC2-1-Eco AAGAATTCTTATTGTATCTTGGCGGC. The forward primer also contained an NdeI restriction site, while the reverse primer contained an EcoRI restriction. The PCR product was ligated into pET30b vector, linearised with the same restriction enzymes. After transformation of BL-21 *E. coli* cells, selection and expression, the protein was obtained as inclusion bodies and was solubilised in 8 M urea and 1 mM DTT. Renaturation was accomplished by extensive dialysis (3 × 12 h) against 50 mM Tris, pH 7.4, containing 0.5 M NaCl. Purification was accomplished by anion-exchange chromatography on DE-52 (Whatman) followed by a second step on Mono-Q (GE-Healthcare), along with standard protocols previously adopted for OBPs and CSPs (Ban et al., 2003; Calvello et al., 2003).

### Fluorescence measurements

Emission fluorescence spectra were recorded on a Horiba scientific Fluoromax-4 spectrofluorometer at room temperature in a right-angle configuration, with a 1 cm light path quartz cuvette and 5 nm slits for both excitation and emission. Proteins were dissolved in 50 mM Tris–HCl buffer, pH 7.4, while ligands were added as 1 mM methanol solutions.

### Ligand-binding experiments

The affinity of HarmNPC2-1 for the fluorescent probe N-phenyl-1-naphthylamine (1-NPN) was evaluated by titrating a 2 μM solution of the protein with aliquots of 1 mM solution of 1-NPN in methanol to final concentrations of 2–16 μM. The probe was excited at 337 nm and emission spectra were recorded between 380 and 450 nm. The dissociation constants of other ligands were measured in competitive binding experiments, where a solution of the protein and 1-NPN, both at the concentration of 2 μM, was titrated with 1 mM methanol solutions of each chemical to final concentrations of 0.5–16 μM. Data obtained with 1-NPN were analyzed with Prism software. Dissociation constants of the competitors were calculated from the corresponding IC_50_ values (the concentration of each ligand halving the initial value of fluorescence), using the equation: K_D_ = [IC_50_]/1+[1-NPN]/K_1−NPN_, [1-NPN] being the free concentration of 1-NPN and K_1−NPN_ being the dissociation constant of the complex Protein/1-NPN.

### Molecular modeling and docking

A three-dimensional model of HarmNPC2-1 was generated using the online programme SWISS MODEL (Peitsch, 1995; Arnold et al., 2006; Kiefer et al., 2009) and the structure of human NPC2 protein (acc. no. 5KWY, identity between the two proteins 35%). Docking was performed by the on-line programme SWISS DOCK using default parameters (Grosdidier et al., 2011). Models were visualized with the UCSF Chimera package. Chimera is developed by the Resource for Biocomputing, Visualization, and Informatics at the University of California, San Francisco (supported by NIGMS P41-GM103311) (Pettersen et al., 2004).

### Western blot analysis

Samples for Western blot analyses were prepared by extracting different tissues with 0.1% TFA and adjusting the concentrations of the extracts to obtain samples with comparable protein concentrations. After electrophoretic separation under denaturing conditions (14% SDS-PAGE), duplicate gels were stained with 0.1% Coomassie blue R250 in 10% acetic acid, 20% ethanol or electroblotted on Trans-Blot nitrocellulose membrane (Bio-Rad Lab) by the procedure of Kyhse-Andersen (1984). After treatment with 2% powdered skimmed milk/Tris overnight, the membrane was incubated with a crude polyclonal antiserum raised against the *Plutella xylostella* orthologue (Pxyl_XP_11568846) of HarmNPC2-1 (custom made in rabbits at the Institute of Genetics and Developmental Biology, Chinese Academy of Sciences, Beijing, China) at a dilution of 1:500 (2 h) and then with goat anti-rabbit IgG horseradish peroxidase conjugate (dilution 1:1,000; 1 h). Immunoreacting bands were detected by treatment with 4-chloro-1-naphthol and hydrogen peroxide.

### *In-situ* hybridization

DIG-labeled antisense and sense probes were synthesized with T7/SP6 DIG RNA Labeling system (Roche, Mannheim, Germany). *In situ* hybridization was performed as described in the reference (Krieger et al., 2002) with some modification. Antennae of 1–2 days old female moths were embedded in Tissue-Tek O.C.T. Compound (Sakura, Torrance, USA) and frozen at −60°C rapidly. Longitudinal sections (10 μm) were prepared with cryostat microtome (LEICA CM1850) at −23°C and pasted on Superfrost Plus slides (Thermo Fisher, New Hampshire, USA). After air-drying at room temperature for about 20 min, the slides were prehybridized along with the following protocol: fixed in 4% paraformaldehyde/0.1 M NaHCO_3_ (pH 9.5) at 4°C for 30 min, then washed with PBS (0.85% NaCl, 1.4 mM KH_2_PO_4_, 8 mM Na_2_HPO_4_, pH 7.1) for 1 min; 0.6% HCl for 10 min; PBS/1% TritonX-100 for 2 min; twice with PBS for 1 min; 50% formamide/5 × SSC (1 × SSC: 0.15 M NaCl, 0.015 M Na-citrate, pH 7.0) for 10 min.

For hybridization, the probes were cut into an average length of about 200 bp with carbonate buffer (80 mM NaHCO_3_, 120 mM Na_2_CO_3_, pH 10.2) (Angerer and Angerer, 1992), then diluted to suitable concentration with hybridization buffer (Boster, Wuhan, China). The slides were incubated with 100 μL hybridization buffer containing antisense/sense probes at 55°C overnight. After two washes with 0.1 × SSC at 60°C for 30 min and a short rinse with TBS (100 mM Tris, pH 7.5, 150 mM NaCl), DIG-labeled RNA probes were detected using DIG Nucleic Acid Detection Kit (Roche, Mannheim, Germany). Finally, slides were treated with 20 μL PBS/Glycerol, covered with coverslips and sealed with nail polish. Sections were observed and images were taken with optical microscope Olympus BX61 (Olympus, Tokyo, Japan). Pictures were adjusted only for brightness or contrast using the cellSens software (Olympus, Tokyo, Japan).

### Immunohistochemistry

Antennae of *H. armigera* were fixed in paraformaldehyde (4%) and glutaraldehyde (2%) in 0.1 M PBS (pH 7.4), then dehydrated in an ethanol series. The samples were embedded in LR White resin and ultrathin sections were cut and mounted on Formvar-coated grids. Sections were stained along with the procedure of Steinbrecht et al. (1992). In brief, preincubation was performed by floating the grids with the sections on solutions of PBG (PBS containing 50 mmol/L glycine) and PBGT (PBS containing 0.2% gelatin, 0.5% bovine serum albumine, and 0.02% Tween-20) twice for each solution. Then, they were incubated in primary polyclonal antiserum against Pxyl_ XP_11568846 (1:1,000) in PBGT overnight at 4°C. After six washings with PBGT, sections were incubated with secondary antibody in PBGT (1:20) (AuroProbeTM EM, GAR G10, Amersham) for 1 h at room temperature, followed by two washings with PBGT, PBG and water. Each washing step was performed with 20 μL droplets for 5 min. Silver intensification (Danscher, 1981) was applied to increase the size of the gold granules and, after six washings with water, it was followed by treatment with 2% uranyl acetate to increase the tissue contrast in transmission electron microscopy. Sections were then observed under a transmission electron microscope (HITACHI H-7500). Pictures were only adjusted for brightness and contrast.

## Results

### NPC2 sequences in lepidoptera

**Figure 1:**
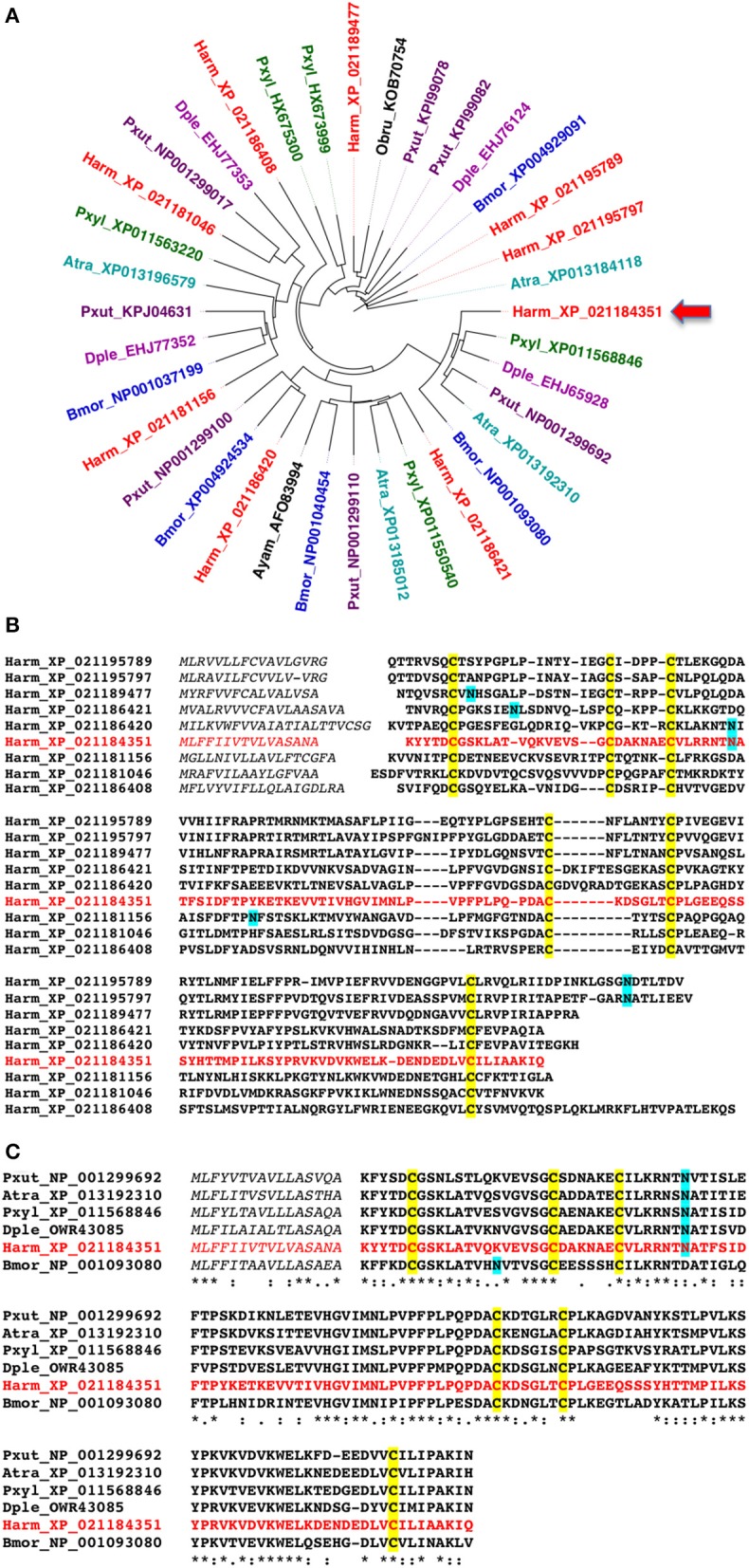
Amino acid sequences of NPC2 proteins of Lepidoptera. **(A)** Phylogenetic tree constructed with the NPC2 protein sequences of some representative Lepidopteran species. Accession numbers are preceded by species abbreviations as follows. Atra: *Amyelois transitella*; Bmor: *Bombyx mori*; Dple: *Danaus plexippus*; Harm: *Helicoverpa armigera*; Pxut: *Papilio xuthus*; Pxyl: *Plutella xylostella*. **(B)** Alignment of the nine amino acid sequences encoded by NPC2 genes in the genome of *H. armigera*. **(C)** Alignment of representative orthologues of HarmNPC2-1, the protein investigated in the present work and indicated by an arrow.

Before starting our experimental work, we searched for NPC2 proteins in Lepidoptera, using the eight sequences of *Bombyx mori* annotated in the NCBI Protein database as templates to blast protein and nucleotide databases. Our search returned a variable number of sequences (4–9) in several lepidopteran species. The phylogenetic tree of Figure 1A is based on sequences of typical species whose genomes have been published, and likely reflect the total number of NPC2 genes present in each species. As compared to OBPs and CSPs, the numbers of NPC2 proteins in Lepidoptera are relatively small, similar to those reported in other orders of insects (4–13) and in some Crustacea and Chelicerata (3–14) (Pelosi et al., 2014). We can also observe that clusters include members from different species, while proteins from the same species fall into different clusters. This fact can be illustrated by the high divergence of the 9 NPC2 sequences of *H. armigera*, which share between each other from 10 to 53% of their amino acids (Figure 1B). By contrast, the orthologous sequences across different species are much more conserved, as shown for one of them in Figure 1C (58–73% identical amino acids).

For our experimental work, we selected Harm_ XP_021184351, indicated in Figure 1A by an arrow and here reported as HarmNPC2-1, to be expressed in a bacterial system. The choice was motivated by the fact that a polyclonal antiserum against the *P. xylostella* orthologue (Pxyl_ XP_11568846) was already available, as part of a parallel on-going study.

Our vector pET30 produced the protein without any modification, apart from an additional methionine at the N-terminus. The protein was expressed in good yield (around 20 mg/L of culture) as insoluble inclusion bodies, was solubilised in urea/DTT and renatured by extensive dialysis against Tris buffer. We observed that the purified recombinant protein was rather unstable in Tris buffer and had a tendency to precipitate during the renaturation dialysis. Eventually we prevented or reduced its aggregation by adding 0.5 M sodium chloride to the dialysis buffer.

### Ligand-binding experiments

We first probed the function of the recombinant protein by measuring its affinity to a series of potential ligands, using a fluorescent reporter and protocols borrowed from ligand-binding assays used with OBPs and CSPs (Ban et al., 2002; Calvello et al., 2003).

**Figure 2:**
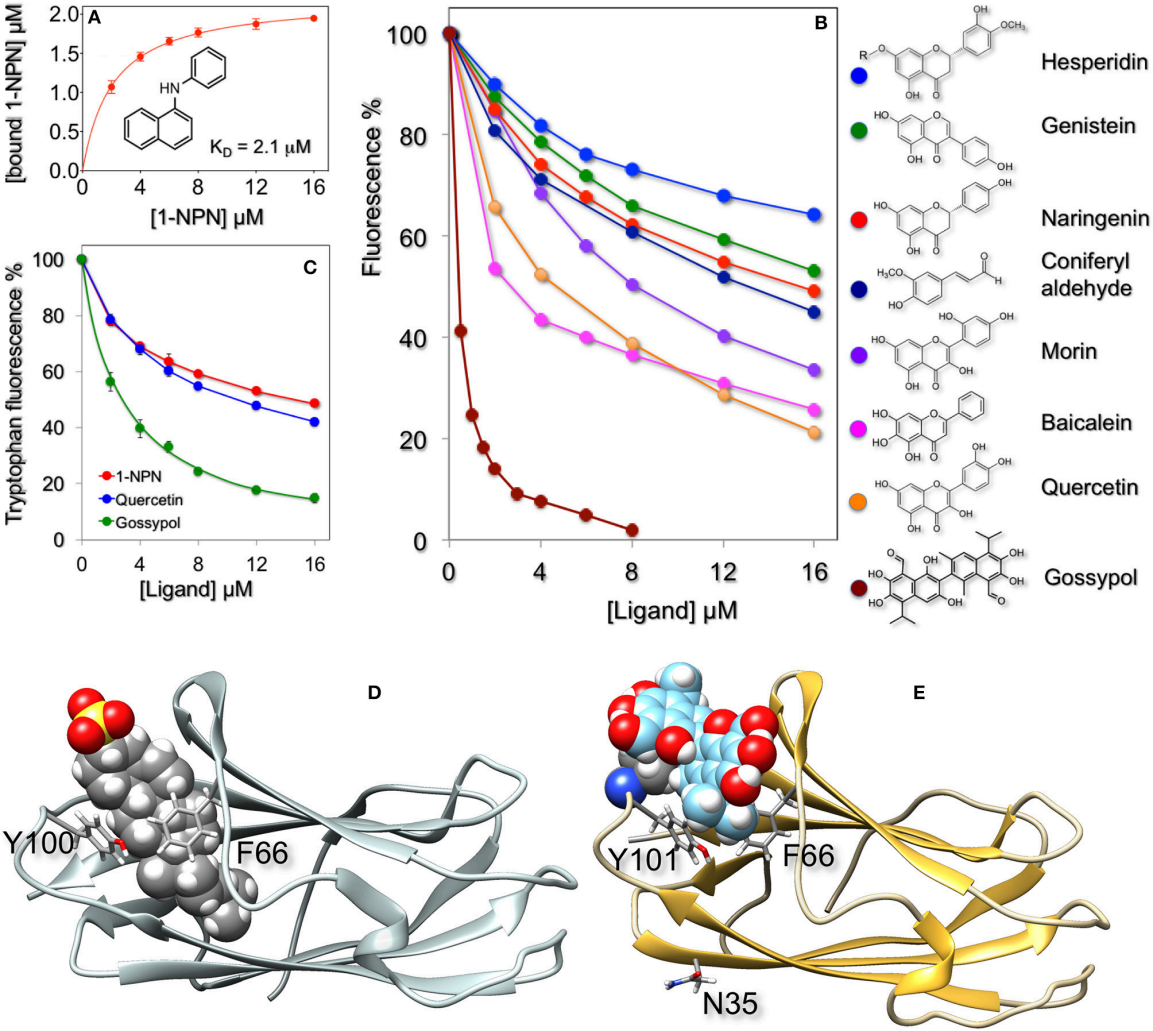
Ligand-binding properties of recombinant HarmNPC2-1. **(A)** The purified protein binds the fluorescent probe 1-NPN with a dissociation constant of 2.1 μM (SEM: 0.14). **(B)** In competitive binding experiments a series of flavonoids exhibited good affinity for HarmNPC2-1. Among natural volatile compounds only coniferyl aldehyde showed moderate binding. Similar affinity was measured with synthetic derivatives (α-methyl, α-methoxy, and α-pentyl) of cinnamaldehyde. The sex pheromone components of *H. armigera* (Z)-11-hexadecenal, (Z)-9-hexadecenal, (Z)-11-hexadecenol and (Z)-11-hexadecenyl acetate, as well as the following chemicals were ineffective in displacing the fluorescent probe from the complex at concentrations up to 16 μM: benzaldehyde, 4-methyl benzaldehyde, 2-hydroxy benzaldehyde, 4-methoxy benzaldehyde, vanillin, cinnamaldehyde, eugenol, piperonyl alcohol, 2-phenylacetaldehyde, safranal, carvone, citronellal, citronellic acid methyl ester, methyl cinnamate, methyl jasmonate, β-ionone, capsaicin, farnesol, indole, β-estradiol, 1-octen-3-ol, octanal, 2,4-octadienal, octanoic acid, decanal, decanoic acid, Z3-hexen-1-ol, 2-hexanol, 3-hexanol, 1-heptanol. **(C)** Quenching of intrinsic tryptophan fluorescence by 1-NPN, quercetin, and gossypol. **(D)** Docking of cholesterol sulfate (shown in fill mode) to bovine NPC2 (PDB: 2HKA) and of gossypol to a model of HarmNPC2-1 **(E)**. In both cases the ligand binds in the same region of the protein and is flanked by the same residues, Phe66 and Tyr100/Tyr101, shown in stick mode. In the model of HarmNPC2-1 the predicted glycosilation site at Asn3 is also shown in stick mode. The model of HarmNPC2-1 was built using the on-line programme SwissModel (Peitsch, 1995; Arnold et al., 2006; Kiefer et al., 2009) and the structure of the human NPC2 (PDB: 5KWY, Li et al., 2016) as template.

The fluorescent probe N-phenyl-1-aminonaphthalene (1-NPN), which binds efficiently to insect OBPs and CSPs, as well as to some vertebrate OBPs, showed good affinity to HarmNPC2-1 with a dissociation constant of 2.1 μM (Figure 2A).

A large number of natural compounds, both pheromones and plant volatiles (all listed in the legend of Figure 2), were used in competitive binding assays. Most of them did not show appreciable affinity, but a few can be regarded as very weak ligands. Among volatile chemicals, only coniferyl aldehyde had moderate affinity with a dissociation constant of 7.4 μM. Other chemicals, reproducing part of this molecule, such as vanillin, capsaicin cinnamaldehyde, eugenol, and piperonyl alcohol, all failed to bind HarmNPC2-1. However, introduction of a methyl, methoxy, or pentyl group in the alpha position of cinnamaldehyde had positive measurable effects on the binding. Therefore, we reasoned that ligands for this protein were molecules of larger size, and so we tested some flavonoids. Figure 2B reports the displacement of 1-NPN from the complex with HarmNPC2-1 by coniferyl aldehyde and some representative flavonoids. The tested compounds proved to be moderate to good ligands, the best was gossypol and the worst was hesperidin. We suspect that the reduced affinity of this last chemical for the protein might be due to the presence of the glycan moiety (indicated with R in the structure), hindering good interaction of the aglycone part with the hydrophobic binding pocket of the protein. Gossypol, the best ligand found so far, is a phytoalexin abundantly present in cotton, one of the main host plants of *H. armigera* (Rajapakse and Walter, 2007). Gossypol contents of 0.4–2% have been reported, depending on tissue and developmental stage (Cai et al., 2004; Tian et al., 2016).

The yellow color associated with gossypol and other flavonoids might lead to doubt about whether the decrease in fluorescence is due to displacement of the probe from the protein, rather than the effect of light absorption. While we cannot entirely dismiss this hypothesis, other observations suggest that our flavonoids interact with the binding pocket of the protein: (a) the binding of quercetin and gossypol to the protein produces quenching of intrinsic tryptophan fluorescence in a dose-dependent fashion similar in behavior and intensity to what is measured with 1-NPN (Figure 2C); (b) if the fluorescence decrease was only due to light absorbance, this phenomenon should have a linear relationship with the concentration of the added ligand; (c) docking simulations suggest that quercetin and gossypol binds HarmNPC2-1 in the same place and with a similar orientation as reported for the binding of cholesterol to vertebrate NPC2 proteins (Xu et al., 2007). Figure 2E reports a model of HarmNPC2-1 complexed with a molecule of gossypol. The ligand sits in the same pocket and is flanked by the same residues (Phe66 and Tyr101) as cholesterol sulfate in the structure of bovine NPC2 (PDB: 2HKA, Figure 2D). Docking of HarmNPC2-1 with quercetin yielded similar results (not shown). We can conclude that light absorption by the yellow pigment might partially affect the observed phenomenon, which is mostly due to competitive binding.

### Immunodetection and *in situ* hybridization

**Figure 3:**
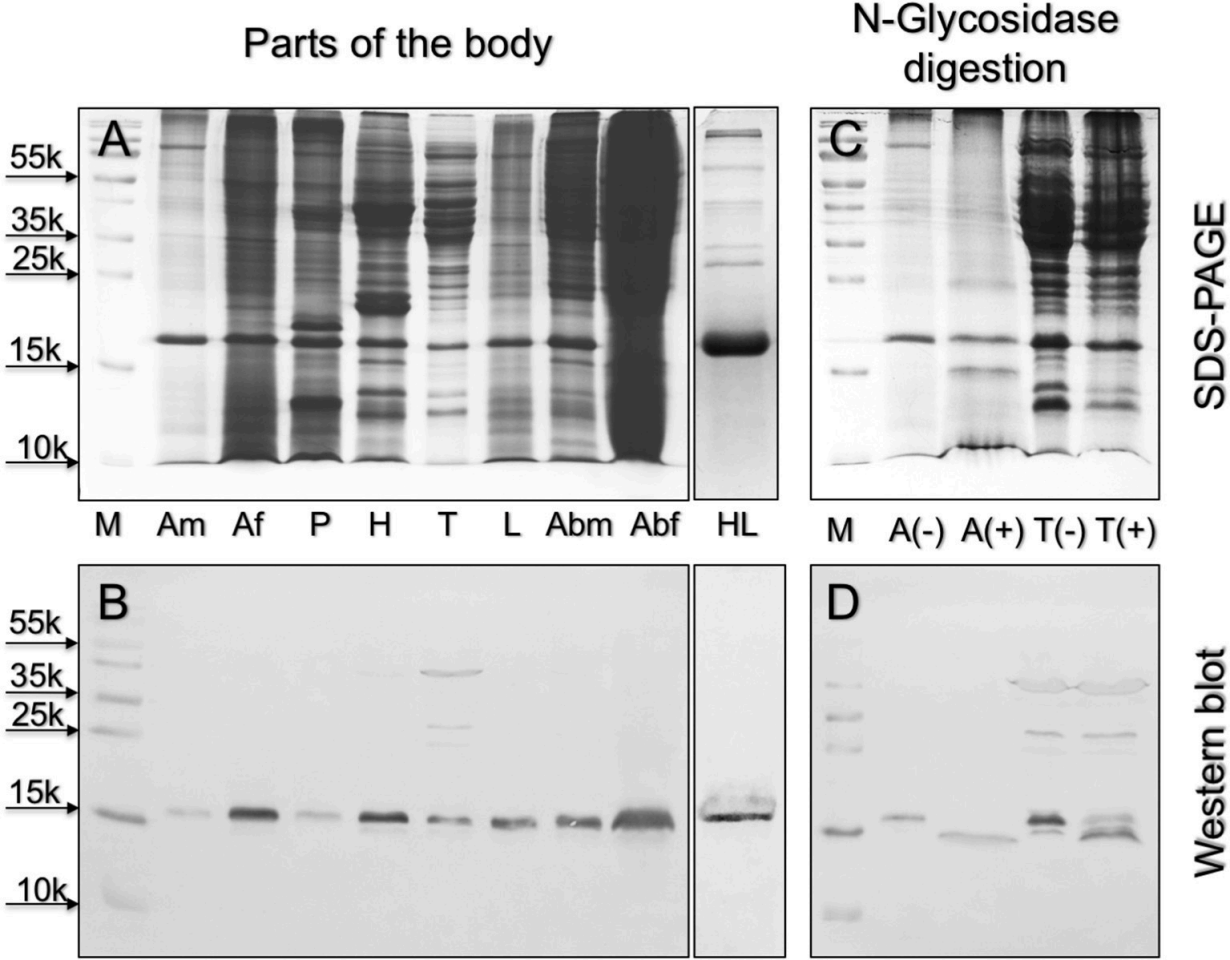
Left: expression of HarmNPC2-1 in different parts of the body of *H. armigera*. **(A)**: Coomassie stained gel; **(B)**: Western blot. The protein is abundant in female antennae, head, legs, and abdomen, but also detectable in male antennae proboscis and thorax. The almost ubiquitous presence of this proteins suggests different functions, as it is often the case with OBPs and CSPs. M, molecular weight markers; Am, male antennae; Af, female antennae; P, proboscis; H, head; T, thorax; L, legs; Abm, male abdomen; Abf, female abdomen; HL, haemolymph. Right: upon digestion with N-glycosidase, the apparent molecular mass of HarmNPC2-1 from antennae (A) and thorax (T), as stained in Western-blot experiments, is shifted from about 16 kDa to about 15 kDa, revealing the presence of N-glycosilation in the native protein. **(C)**: Coomassie stained gel; **(D)**: Western blot; (–) and (+) indicate samples before and after enzyme treatment, respectively.

The hypothesis that flavonoids might represent the natural ligands for HarmNPC2-1 carries an important consequence at the physiological level. These compounds are non-volatile and can only be detected through contact chemodetection; therefore, HarmNPC2-1 is likely to be associated with taste sensilla. To probe this hypothesis, we used Western blot analysis and monitored the tissue expression of the protein. Figure 3 reports the results obtained with the antiserum raised against the *P. xylostella* orthologue of our protein (identity between the two mature sequences is 67% at the amino acid level). Although expressed at different levels in the parts of the body examined, our protein seems to be rather ubiquitous, a fact that could suggest more than one function. Incidentally, we can observe that also OBPs and CSPs have been reported to be expressed in different organs, a fact related to their multiple physiological roles (Pelosi et al., 2017). In particular, we can notice that females have higher levels of HarmNPC2-1 in their antennae compared to males, while only traces are detected in the proboscis. Good expression has been also observed in the head, thorax, and legs, as well as in the abdomen of both sexes. The haemolymph also contains HarmNPC2-1, a fact that could be in part responsible for the ubiquitous detection of the protein. Cross reactivity with other members of the same family can be excluded as HarmNPC2-1 shows 10–23% identity with any of the other 8 NPC2 proteins identified in the genome of *H. armigera*.

Using our antiserum, we have also verified that HarmNPC2-1 is N-glycosilated. In both the antennae and thorax, the apparent molecular weight of the protein is shifted from about 16 kDa to <15 kDa after treatment with N-glycosidase; the predicted molecular weight of the mature, unmodified protein is 14,482 Da. On the other hand, a potential site of glycosilation is predicted at Asn35 (Figure 1C). As suspected, this residue is exposed to solvent in the model of the protein (Figure 2E). It is interesting to observe that a potential glycosylation site is conserved in most of the orthologous sequences, while it is located in different regions of the amino acid sequence in the other NPC2 of the same species (Figure 1B). The presence of a glycan region linked to the polypeptide chain can account for the good solubility of the native protein, as opposed to the ease of aggregation exhibited by the recombinant HarmNPC2-1.

**Figure 4:**
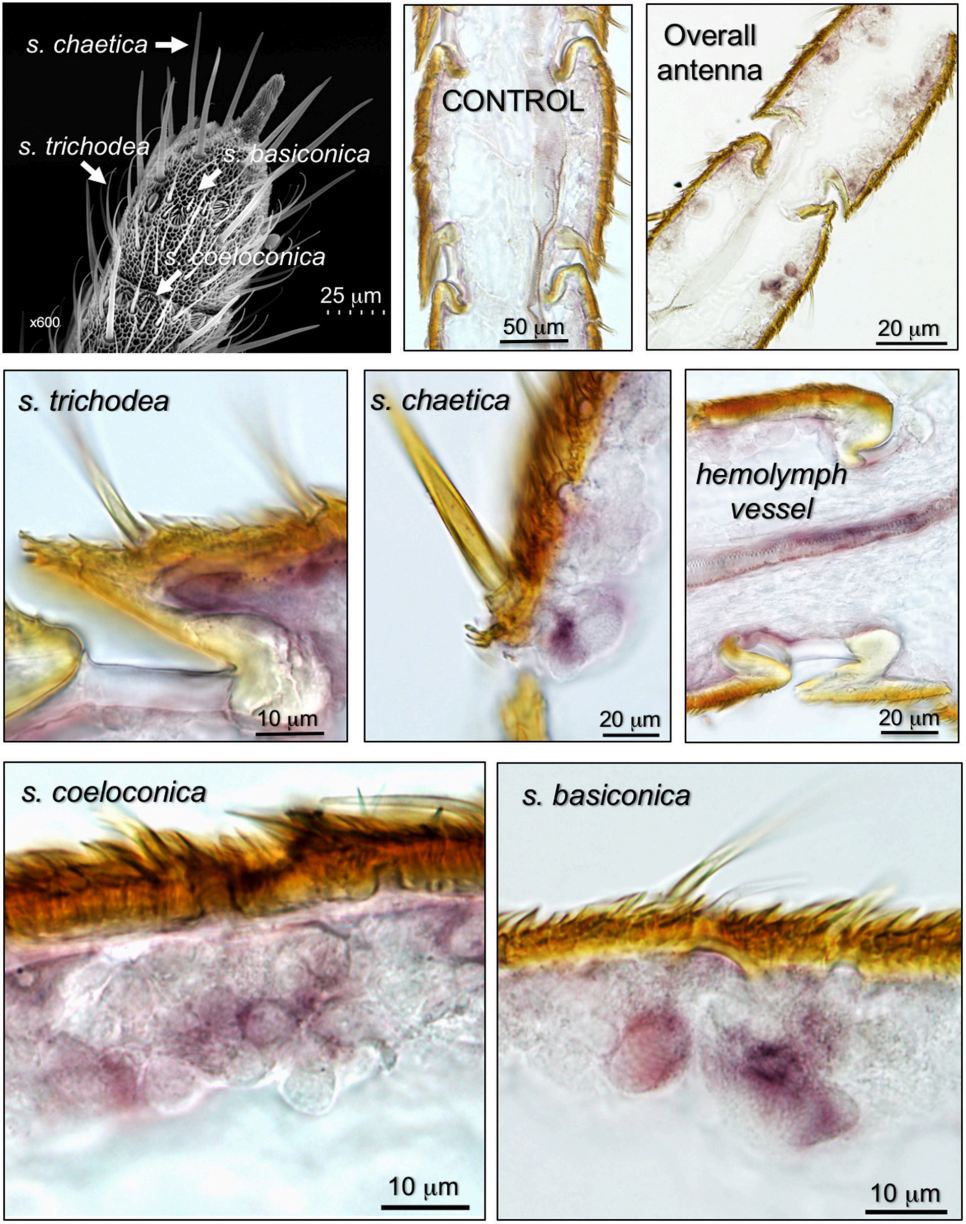
Expression of the gene encoding HarmNPC2-1 in the antenna of *H. armigera*, detected by *in-situ* hybridization. The top-left panel shows an SEM picture of the tip of the antenna, where different types of chemosensilla are indicated. The other panels show that the gene encoding HarmNPC2-1 is abundantly present in cells at the base of sensilla trichodea, basiconica, coeloconica, and chaetica, likely the same types of cells involved in the synthesis and degradation of OBPs and CSPs. Heavy staining was also detected in hemolymph vessels, a result predicted by Western blot experiment and partly justifying the ubiquitous presence of HarmNPC2-1 in the insect body.

We then focused our attention at the sensillum level and used female antennae, which seem to express larger amounts of NPC2-1, to monitor the expression of the gene, using *in situ* hybridization experiments. The results (Figure 4) revealed the presence of the gene encoding HarmNPC2-1 in all types of sensilla: trichodea, basiconica, coeloconica, and chaetica, with the last type probably being the preferential site of expression. Strong staining is visible in cells at the base of the sensilla, likely the same kinds of cells that are involved in synthesizing and recycling OBPs and CSPs.

**Figure 5:**
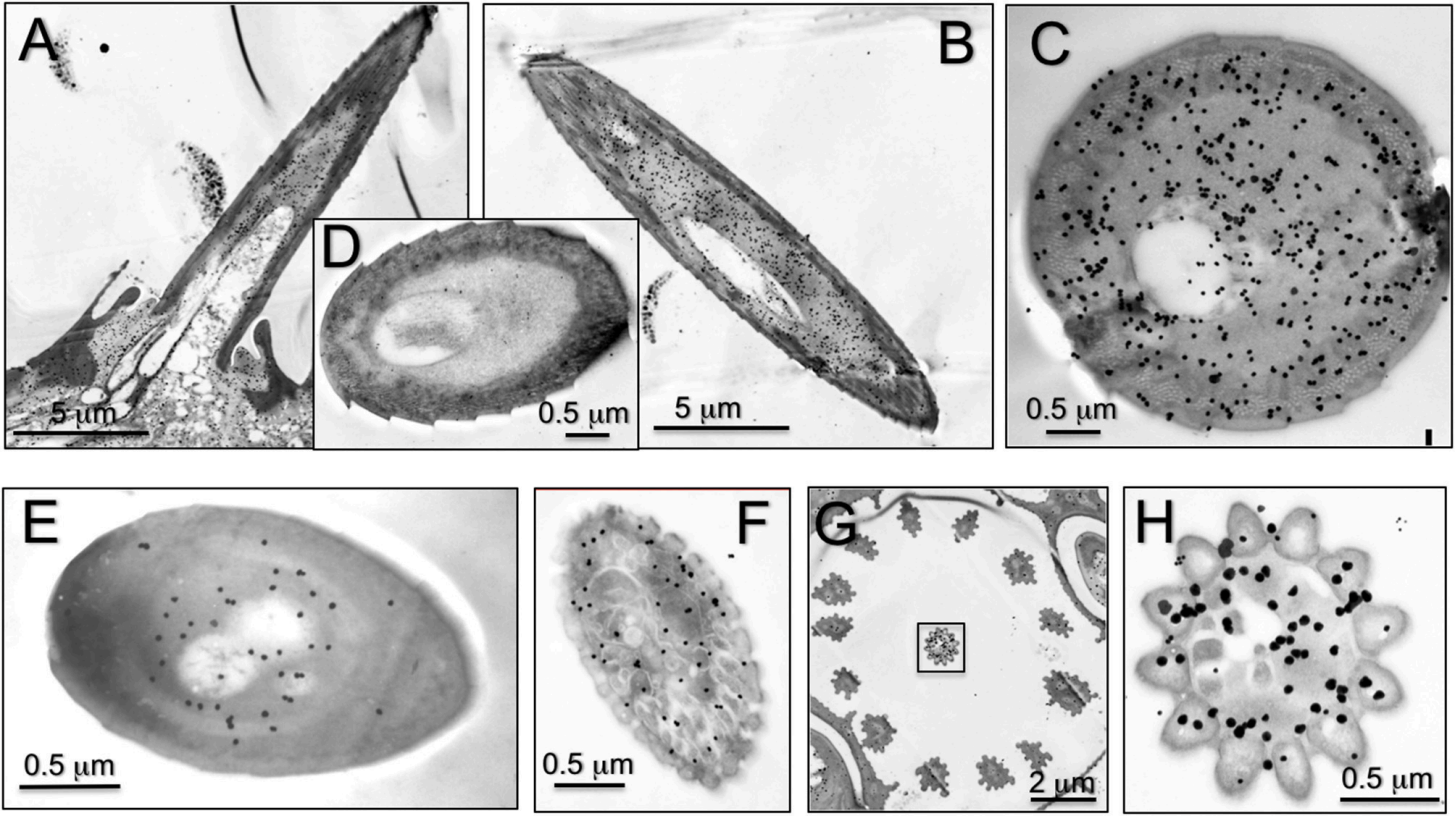
Immunolocalization of HarmNPC2-1 in adult antennae of *H. armigera*. Staining in cross-sections of different sensilla was observed. **(A)** Longitudinal section of sensilla chaetica; **(B,C)** cross section of sensilla chaetica; **(E)** trichodea; **(F)** basiconica; **(G,H)** coeloconica. In particular, NPC2-1 is much more concentrated in sensilla chaetica and strong staining is also visible at the base of the sensilla **(A)**. **(D)** Shows a sensillum chaeticum treated as control by omitting the first antiserum.

We then complemented the data on RNA localisation with immunocytochemistry to stain the expressed proteins. Representative results are reported in Figure 5. We detected some staining in sensilla trichodea, basiconica, and coeloconica, but the strongest labeling was observed in long sensilla chaetica. As with OBPs and CSPs, this protein is present in the sensillar lymph, suggesting roles of transport similar to those performed by the other two classes of carrier proteins.

## Discussion

Aim of this study was to shed light on the physiological functions of NPC2 proteins in arthropods. While in non-insect arthropods NPC2 proteins could act as semiochemical carriers, in the absence of other suitable candidates, we wondered whether in insects these proteins could share the same function with OBPs and CSPs. To support such hypothesis we need to verify that NPC2 proteins (1) are expressed in the lymph of chemosensory structures and (2) bind odorants and pheromones with some degree of specificity and affinity similar to those of OBPs and CSPs.

In the present study we have shown that one of the nine NPC2 proteins predicted by the genome in *H. armigera* is abundantly expressed in all types of chemosensory hairs: the gene is detected in cells at the base of the sensilla, likely the same types of cells dedicated to synthesis and recycling of OBPs and CSPs, and the expressed protein fills the sensillar lymph. Moreover, HarmNPC2-1, and likely other members of the same family, is present not only in chemosensory organs, but also in the abdomen and other parts of the body, as well as in the haemolymph. Such wide expression across the body is an aspect shared with OBPs and CSPs, which are involved not only in the detection, but also in the delivery of semiochemicals, as well as in roles completely unrelated to chemical communication (Pelosi et al., 2017). In particular, the high levels of proteins detected in the abdomen of both sexes is reminiscent of OBPs and CSPs reported in reproductive organs, where they have been suggested to act as pheromone carriers (Sun et al., 2012; Ban et al., 2013; Zhou et al., 2013). In addition, NPC2 proteins could be involved in contrasting the toxic effects of gossypol by tightly binding and sequestrating this molecule. Such role would be most important in the larvae in case high levels of NPC2 proteins were found at this developmental stage. It has been shown that UDP-glycosyltransferases is involved in detoxification from gossypol in Heliothine larvae, based on the large proportion of this chemical found in glycosylated form in the feces (Krempl et al., 2016a,b), but other mechanisms could also be active.

The fact that HarmNPC2-1 is tuned to non-volatile ligands fits with its abundant expression in sensilla chaetica, which are dedicated to contact chemoreception. However, the presence and function of the same protein in other types of chemosensilla has still to be clarified. While proteins of the NPC2 family likely complement the function of OBPs and CSPs in insects, in other arthropods they could represent the only class of soluble proteins involved in chemical communication. In fact, OBPs are absent outside of Hexapoda, while the presence of only one or two members of CSPs in Crustacea does not seem to support a role in chemodetection (Pelosi et al., 2014, 2017). The involvement of NPC2 proteins in chemical communication in insects, as well as in other arthropods, adds new targets in environmental-friendly strategies for pest population control.

## Author contributions

GW and PP: designed research; JZ, MG, L-MS, and LB: performed research; all authors analyzed data and discussed results during the progress of the work; GW, YL, and LB: contributed biological samples, reagents, analytical tools, and laboratory equipments; PP and GW: wrote the paper with contributions from MG and LB. All authors gave final approval for publication.

### Conflict of interest statement

The authors declare that the research was conducted in the absence of any commercial or financial relationships that could be construed as a potential conflict of interest.
